# Negative Emotions Are Associated With Older Self-perceived Age: A Cross-section Study From the UK Biobank

**DOI:** 10.34172/ijhpm.8060

**Published:** 2024-07-09

**Authors:** Tianyi Wang, Shixing Feng, Junqi Wang, Hangyu Li, Yang Song, Dongran Han, Yixing Liu

**Affiliations:** ^1^School of Management, Beijing University of Chinese Medicine, Beijing, China; ^2^Department of Neurology, Dongfang Hospital Beijing University of Chinese Medicine, Beijing, China; ^3^Dongzhimen Hospital Beijing University of Chinese Medicine, Beijing, China; ^4^School of Life and Science, Beijing University of Chinese Medicine, Beijing, China; ^5^School of Humanities, Beijing University of Chinese Medicine, Beijing, China

**Keywords:** Negative Emotions, Aging, Self-Perceived Age, Multinomial Logistic Regression, UK Biobank

## Abstract

**Background:**

Prior research has indicated a potential connection between psychological stress and how individuals perceive their own age. Building on this foundation, the current study explores the relationship between negative emotions and self-perceived age.

**Methods:**

We conducted a cross-sectional analysis using data from the UK Biobank, a comprehensive cohort study representing the UK population. The analysis included 347 892 participants, aged between 39 and 73 years, of which 184 765 were women, accounting for 53.1% of the sample. Participants were categorized into three groups based on their self-perceived age: feeling younger than their chronological age (group Younger), feeling older than their chronological age (group Older), and feeling as old as their actual age (group Same). To investigate the relationship between negative emotions and self-perceived age, we utilized a multinomial logistic regression model with the Younger group serving as the reference category.

**Results:**

Of 347 892 participants, after adjusted for covariates, the results showed that participants with irritability, nervous feelings, worrier/anxious feelings or fed-up feelings, worry too long and loneliness/isolation are more likely to be rated as "about your age" or "older than you are," with "younger than you are" as the reference group, indicating that negative emotions may influence one’s self-perceived age. Among those negative emotions, irritability has the most significant impact self-perceived age, with the odds ratios (ORs) being 1.44 (95% CI: 1.35-1.54) and 1.11 (95% CI: 1.09-1.14).

**Conclusion:**

Negative emotions are associated with older self-perceived age, and irritability has the greatest impact. Further studies analyzing self-perceived age are needed to take psychological factors into consideration.

## Background

**Key Messages**

**Implications for policy makers**

Practical recommendations for policy-makers include the following:

- This article suggests that negative emotions are an essential factor influencing individuals’ self-perceived age. Government and public health departments should prioritize mental health education to raise awareness about the adverse effects of negative emotions.
- In addition to maintaining a healthy psychological state, scientific and healthy lifestyles are equally significant in slowing ageing. This includes practices such as sun protection, quitting smoking and alcohol consumption, ensuring an adequate amount of sleep, as well as engaging in regular physical exercise.
- In shaping future policies, particular attention should be given to mental health considerations. Policy-makers should focus on enhancing the mental health system and allocating necessary resources, including staffing, funding, and materials, to promote overall mental well-being in the population.

**Implications for the public**

The World Health Organization (WHO) proposes that a complete health state also includes a healthy psychological state. However, in today’s rapidly developing society, everyone must face pressure from all sides, which makes more and more people deeply in negative emotions every day, making the health status receive a significant impact, not only may accelerate the aging mentioned in this study, but also may produce a series of problems. Each of us is responsible for our own health, so it is essential and urgent for the public to learn to regulate their own emotional problems. It is important to seek help from family, friends, teachers or doctors in a timely and courageous manner to relieve internal stress and maintain a healthy mental state.

Self-perceived age is usually the estimation rating of people’s actual age, a measure of how one perceives one’s own aging process. Lawton employed subjective age felt by the elderly themselves to assess themselves in 1975.^1^ People tend to be asked that “How do you think that you look like?” and “younger,” “older,” or “same as your actual (biological/chronological) age” are the main three answers.^2^ As part of assessment for a person, it has been used as an indicator for health status, even one’s consumption behavior.^1,3,4^ Several studies have point it out that younger age,^5^ low socioeconomic status,^6^ gender,^7^ unhealthy marriage,^4,6^ and poor self-rated health^8^ are the main influential factors for older self-perceived age. What’s more, because this assessment usually performed by individual’s face (sometimes may be the neck or the back of hand),^9^ the changes in one’s appearance may also affect the judgement for self-perceived age, like wrinkles, increased pigmentation, loss of elasticity and firmness, and dull skin,^10^ which are the signs of facial aging.Besides, air pollution,^11,12^ sun light (ultraviolet),^13^ smoking,^14^ sleep,^15^ and body mass index (BMI)^16^ will also accelerate one’s facial aging, which indirectly influences the self-perceived age. Research showed that loneliness and depressive symptoms are associated with older self-perceived age, indicating that mental or psychological stress also plays an important role in self-perceived age.^6^ However, few studies have investigated this link.

 In psychological discourse, a clear distinction has been made between “psychological stress”—a persistent response to life’s pressures encompassing a range of emotional experiences—and “negative emotions,” which refer specifically to transient states such as anger, sadness, and anxiety. Unlike psychological stress, which may have chronic implications, negative emotions are acute responses that, despite their brevity, significantly influence an individual’s perception of their own aging process.

 Studies have found that negative emotions are associated with mental conditions, indigestion, poor sleep and one’s interpersonal relations and work efficiency.^17-20^ What’s more, researchers also revealed that negative emotions cause the body to produce more stress hormones, such as cortisol and adrenaline, which accelerate cell ageing and reduce immune system function, thereby increasing the risk of disease,^18^ indicating negative emotions may serve as a catalyst accelerating ageing. Therefore, the first hypothesis was made in this research that negative emotions are associated with older self-perceived age. A bodily maps of emotions depicted participants’ bodily feeling experiencing different kinds of negative emotions, revealed the differences between negative and positive emotions, and within negative emotions.^21^ Therefore, the second hypothesis was that different types of negative emotions may affect people differently.

 The study data comes from UK Biobank, a large population-based cohort that allows important lifestyle behaviours to be measured at the same time for a well-described cohort.^22^ It was expected that negative emotions might make a person look older than his or her actual age, and different types of negative emotions might have different influences on self-perceived age.

## Methods

###  Study Population 

 UK Biobank is a large, ongoing prospective cohort study.^23^ The data of this study was from the baseline measures of UK Biobank occurring between 2006 and 2010, via 22 assessment centres across the United Kingdom, which recruited 502 468 participants aged from 37 to 73 in England, Scotland and Wales.^24^ There are six phases during each visit to an assessment centre: consent, touchscreen questionnaire (ie, demographic information, occupation, lifestyle, early life exposures, cognitive function, family history, and medical history), verbal interview, eye measures, physical measures, and blood/urine sample collection. The variables used for this study were collected from the “Lifestyle and environment” section of the “Touchscreen questionnaire” at the baseline survey. Participants will touch the screen and choose one of the listing options optimal for themselves. The National Information Governance Board for Health and Social Care and the National Health Service Northwest Multi-center Research Ethics Committee approved this programme and all participants provided electronically signed consent prior to data collection.

**Figure 1:**
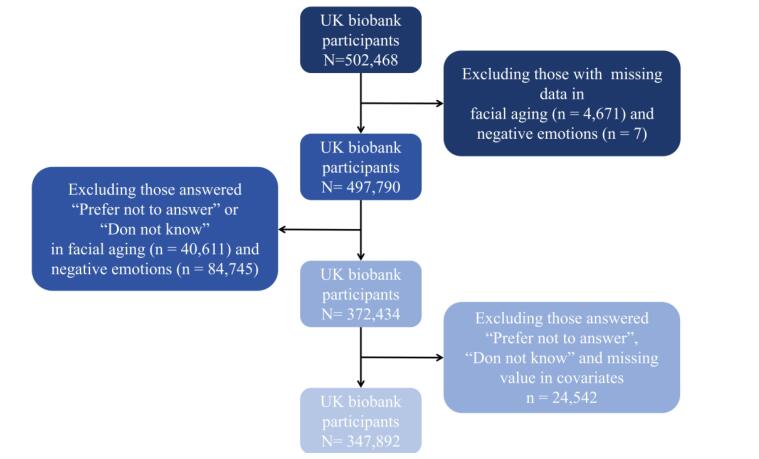
Flowchart of Data Filtering.

 The exclusion criteria were applied as follows: (1) participants with missing value on variable of facial aging were excluded. (n = 4671); (2) participants with missing value on emotional variables were excluded (n = 7); (3) participants who answered “Prefer not to answer” and “Do not know” in facial aging (n = 40 611) or emotional variables were further excluded duo to those non-valuable information (n = 84 745); (4) participants with missing value on covariates (n = 24 542). Finally, a total of 347 892 participants with complete data sets were included in the complete case analysis. Among them, 257 723 participants were in the group of “younger than you are” (group Younger), 7137 were in the group of “older than you are” (group Older), and 83 032 participants were in the group of “about your age” (group Same) (Figure 1).

###  Exposure Assessment

 Participants were asked “Do people say that you look?,” and they could choose from several options: (1) younger than you are, (2) older than you are, (3) about your age, (4) Do not know, and (5) Prefer not to answer. We used this question as an outcome variable for our study.

 The emotional variables came from thirteen items in the mental health questionnaire, including (1) Does your mood often go up and down?; (2) Do you ever feel “just miserable” for no reason?; (3) Are you an irritable person?; (4) Are your feelings easily hurt?; (5) Do you often feel “fed-up”?; (6) Would you call yourself a nervous person?; (7) Are you a worrier?; (8) Would you call yourself tense or “highly strung”?; (9) Do you worry too long after an embarrassing experience?; (10) Do you suffer from “nerves”?; (11) Do you often feel lonely?; (12) Are you often troubled by feelings of guilt?; and (13) Would you describe yourself as someone who takes risks? The options for these questions were “Yes,” “No,” “Do not know,” and “Prefer not to answer.”

###  Socio-demographic Variables 

 The first part of covariates was the demographic information, including gender (male; female); age at recruitment (39-49 years; 50-59 years; or 60-73 years); social and economic status (measured using Townsend deprivation index, with a higher value indicating a lower socioeconomic status for the individual; and categorized into four categories using the 25 quantile, median, and 75 quantile); education level (university degree or equivalent and above; or high school degree or equivalent and below); current employment status (employed and unemployed); race background (White; Mixed; Asian or Asian British; Black or Black British; Chinese; and other ethnic groups).

###  Lifestyle Variables

 The second part of covariates was lifestyle information, including BMI (kg/m^2^, <24.9 kg/m^2^; 25-29.9 kg/m^2^; ≥30 kg/m^2^),^25^ smoking status (never; previous; or current); alcohol intake frequency (daily or almost daily; three or four times a week; once or twice a week; once to three times a month; special occasions only; and never); physical activity level (min/wk, the total time of physical activities were categorized into four categories using the 25 quantiles, median, and 75 quantiles), sleep duration (<7 hours; 7-8 hours; or >8 hours)^26^; time spent outside in summer, and time spent outside in winter.

###  Statistical Analysis

 All the analyses were performed using STATA statistical software 17 (Stata Corp., TX, USA). Individuals with missing data (the options of “Do not know” or “Prefer not to answer” were treated as missing values) on perceived age and emotional variables were excluded (Figure 1). Continuous variables are expressed as median and interquartile range and categorical variables are described as the sample size (n) and percentage (%). Then, Kruskal–Wallis test and chi-square test are used to determine whether there were statistical differences among different groups (group Younger, group Same, and group Older). We assumed that the dependent variable is an ordered variable (older > same > younger), therefore the assumption of proportional odds was assessed first. If this assumption was satisfied, the ordinal logistic regression would be conducted; otherwise, multinomial logistic regression would be performed.

 There were three models tested in this research: Model 1 (adjusted for sex and age); Model 2 (adjusted for all the demographic covariates); Model 3 (adjusted for all the demographic and lifestyle covariates). More detail about descriptions of reference level for the nominal or ordinal covariates were summarized below: age (reference: 39-49); sex (reference: Female); BMI (reference: <24.9 kg/m^2^); Townsend deprivation index (reference: least deprived (Q1)); ethnicity (reference: White/British); sleep duration (reference: <7 hours); alcohol (reference: Daily or almost daily); smoking (reference: Never); metabolic equivalent (MET) score (reference: <396). A two-sided *P* < .05 was considered statistically significant.

## Results

 The characteristics of participants are summarized in Table 1. A total of 347 892 participants were included in this study. Participants were grouped into three groups according to their self-perceived age. A total of 257 723 individuals reported a younger self-perceived age, 7137 reported older than you are and 83 032 chose about your age.

**Table 1:** Characteristics of Participants in Three Groups (n = 347 892)

	**Facial Ageing**			***P*** ** Value**
	**Younger Than You Are** **(n = 257 723)**	**Older Than You Are** **(n = 7137)**	**About Your Age** **(n = 83 032)**	
Sex, No. (%)				<.001
Female	145 272 (56.4)	1457 (20.7)	38 036 (45.8)	
Male	112 451 (43.6)	5662 (79.3)	44 996 (54.2)	
Age at recruitment, No. (%)				<.001
39-49	59 945 (23.3)	2736 (38.3)	21 315 (25.7)	
50-59	86 832 (33.7)	2739 (38.4)	27 892 (33.6)	
60-73	110 946 (43.0)	1662 (23.3)	33 825 (40.7)	
Townsend deprivation index, No. (%)				<.001
Q1	66 304 (25.7)	1635 (22.9)	22 790 (27.4)	
Q2	65 664 (25.5)	1676 (23.5)	21 985 (26.5)	
Q3	64 974 (25.2)	1715 (24.0)	20 682 (24.9)	
Q4	60 781 (23.6)	2111 (29.6)	17 575 (21.2)	
Qualifications, No. (%)				<.001
College or university degree	89 071 (34.6)	2337 (32.7)	27 806 (33.5)	
Other academic qualifications	168 652 (65.4)	4800 (67.3)	55 226 (66.5)	
Current employment status, No. (%)				<.001
In employment	156 099 (60.6)	4613 (64.6)	47 863 (57.6)	
Unemployment	101 624 (39.4)	2524 (35.4)	35 169 (42.4)	
Ethnic background, No. (%)				<.001
White/British	235 577 (91.4)	6397 (89.6)	77 457 (93.3)	
Mixed	8825 (3.4)	384 (5.4)	2789 (3.4)	
Asian	9264 (3.6)	222 (3.1)	2131 (2.6)	
Black African	1309 (0.5)	39 (0.5)	234 (0.3)	
Chinese	763 (0.3)	16 (0.2)	103 (0.1)	
Other	1985 (0.8)	79 (1.1)	318 (0.4)	
BMI, No. (%)				<.001
<24.9	87 094 (33.8)	1475 (20.7)	23 638 (28.5)	
25–29.9	11 2347 (43.6)	2870 (40.2)	35 881 (43.2)	
≥30	58 282 (22.6)	2792 (39.1)	23 513 (28.3)	
Sleep duration, No. (%)				<.001
<7 h	61 122 (23.7)	2192 (30.7)	19 085 (23.0)	
7–8 h	177 294 (68.8)	4289 (60.1)	57 473 (69.2)	
>8 h	19 307 (7.5)	656 (9.2)	6474 (7.8)	
Smoking status, No. (%)				<.001
Never	143 213 (55.6)	3337 (46.8)	44 339 (53.4)	
Previous	89 983 (34.9)	2382 (33.4)	29 363 (35.4)	
Current	24 527 (9.5)	1418 (19.9)	9330 (11.2)	
Alcohol intake frequency, No. (%)				<.001
Daily or almost daily	54 550 (21.2)	1517 (21.3)	18 274 (22.0)	
Three or four times a week	61 387 (23.8)	1539 (21.6)	20 234 (24.4)	
Once or twice a week	67 606 (26.2)	1748 (24.5)	21 156 (25.5)	
One to three times a month	28 756 (11.2)	726 (10.2)	8721 (10.5)	
Special occasions only	27 532 (10.7)	816 (11.4)	8442 (10.2)	
Never	17 892 (6.9)	791 (11.1)	6205 (7.5)	
Total MET minutes, No. (%)				<.001
<396	57 481 (22.3)	1982 (27.8)	19 335 (23.3)	
396-1398	64 985 (25.2)	1909 (26.7)	22 529 (27.1)	
1398-3132	66 948 (26.0)	1592 (22.3)	21 272 (25.6)	
>3132	68 309 (26.5)	1654 (23.2)	19 896 (24.0)	
Time spent outdoors in summer, median (25th, 75th)	3.00 [2.00, 5.00]	3.00 [2.00, 5.00]	3.00 [2.00, 5.00]	<.001
Time spent outdoors in winter, median (25th, 75th)	1.00 [1.00, 2.00]	1.00 [1.00, 3.00]	1.00 [1.00, 2.00]	.494

Abbreviations: BMI, body mass index; MET, metabolic equivalent.

 Gender, age, Townsend deprivation index, income, employment status, BMI, smoking status, and alcohol consumption are statistically different in three groups. Individuals who reported “younger than you are” were more likely to be female and high economic status and employed. In terms of lifestyle factors, people reporting a younger self-perceived age had a lower percentage of obesity (BMI ≥ 30), a high percentage of 7-8 hours sleep. What’s more, they smoked less, and did more sports. Time spent outdoor in winter were not statistically significant. Due to a majority of White/British, the race discrepancy will not be discussed in here.

 The statistical description for emotional variables was described in Table 2. Compared with those chose “younger than you are,” participants reported “Olde than you are” had a high percentage in all emotional variables. Mood swings, miserableness, sensitivity/hurt feelings, fed-up feelings, worrier/anxious feelings and worry too long are the most common emotions. More than 50% of participants reported those negative emotions. Participants in the two groups showed a large difference in irritability, with 26.3% of participants in group Younger reporting this while this number being 43.0% in group Older. Individuals reported “older than you are” experienced more negative emotions, followed by those chose “about your age.” People who chose “younger than you are” experienced fewer negative emotions.

**Table 2:** Core Variables (Including 13 Variables in “Mental Health” Field) of Three Groups (n = 347 892)

	**Facial Ageing**			***P*** ** Value**
	**Younger Than You Are** **(n = 257 723)**	**Older Than You Are** **(n = 7137)**	**About Your Age** **(n = 83 032)**	
Mood swings, No. (%)				<.001
Yes	109 326 (42.4)	4027 (56.4)	36 245 (43.7)	
Miserableness, No. (%)				<.001
Yes	105 675 (41.0)	3595 (50.4)	33 928 (40.9)	
Irritability, No. (%)				<.001
Yes	67 704 (26.3)	3068 (43.0)	24 321 (29.3)	
Sensitivity/hurt feelings, No. (%)				<.001
Yes	140 614 (54.6)	3911 (54.8)	42 493 (51.2)	
Fed-up feelings, No. (%)				<.001
Yes	97 142 (37.7)	3727 (52.2)	32 730 (39.4)	
Nervous feelings, No. (%)				<.001
Yes	55 612 (21.6)	2060 (28.9)	18 700 (22.5)	
Worrier/anxious feelings, No. (%)				<.001
Yes	137 804 (53.5)	4229 (59.3)	45 131 (54.4)	
Tense/highly strung, No. (%)				<.001
Yes	43 317 (16.8)	1831 (25.7)	13 892 (16.7)	
Worry too long, No. (%)				<.001
Yes	117 076 (45.4)	3592 (50.3)	37 903 (45.6)	
Suffer from nerves, No. (%)				<.001
Yes	51 674 (20.1)	2121 (29.7)	17 284 (20.8)	
Loneliness, isolation, No. (%)				<.001
Yes	45 737 (17.7)	1856 (26.0)	13 287 (16.0)	
Guilty feelings, No. (%)				<.001
Yes	72 618 (28.2)	2365 (33.1)	22 552 (27.2)	
Risk-taking, No. (%)				<.001
Yes	75 076 (29.1)	2493 (34.9)	20 993 (25.3)	

 The parallelism tests’ result is summarized in Table S1, indicating the models did not satisfy the proportional odds assumption. Therefore, multinomial logistic regression analysis was performed. In addition, given that the number of variables included in the model, variance inflation factor (VIF) or tolerance were tested to prevent multicollinearity. A VIF >10 or a tolerance <0.1 was regarded as multicollinearity. The results showed no multicollinearity among the variables (Table S2).

**Figure 2:**
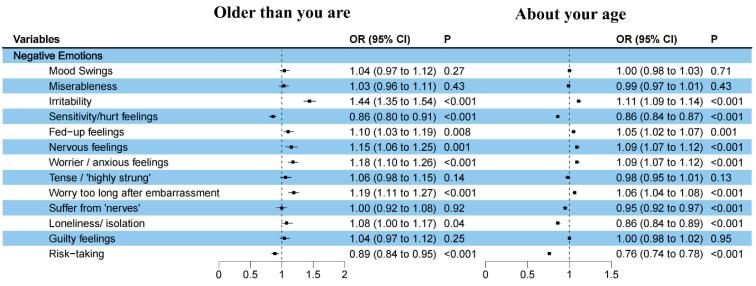
Association Between Self-perceived Age and Negative Emotions Adjusted by Age, Gender, Townsend Deprivation Index, Qualification, Employment Status, BMI, Sleep Duration, Smoking, Alcohol Consumption, MET Minutes, Time Spent Outdoors in Summer, and Time Spent Outdoors in Winter. Individuals reporting “younger than you are” served as the reference group. Abbreviations: BMI, body mass index; MET: metabolic equivalent; OR, odds ratio; CI, confidence interval.

Figure 2 presents the odds ratios (ORs) and 95% confidence interval (CI) of negative emotions from multinomial logistic regression models after adjusted for all covariates. Their tabular results were summarized in Table S3. Covariates’ ORs and 95% CI were summarized in Table S4. Participants reporting “younger than you are” served as the reference group. The results showed that, after adjusted for covariates, experiencing of irritability, fed-up feelings, nervous feelings, worrier/anxious feelings, worry too long after embarrassment, loneliness/isolation had statistically significant associations with a self-perceived age of “older than you are.” Among those negative emotions, irritability had the highest impact on “older than you are” self-perceived age rating, with its OR was 1.44 (95% CI: 1.35 to 1.54), followed by worry too long after embarrassment (OR: 1.19, 95% CI: 1.11 to 1.27), worrier/anxious feelings (OR: 1.18, 95% CI: 1.10 to 1.26) and nervous feelings (OR: 1.15, 95% CI: 1.06 to 1.25) Irritability, fed-up feelings nervous feelings, worrier/anxious feelings and worry too long after embarrassment have statistically significant association with a self-perceived age of “about your age.” Among those negative emotions, irritability also had the highest impact on it, with its OR was 1.11 (95% CI: 1.09 to 1.14), followed by worry too long (OR: 1.06, 95% CI: 1.04 to 1.08), worrier/anxious feelings (OR: 1.09, 95% CI: 1.07 to 1.12) and nervous feelings (OR: 1.09, 95% CI: 1.07 to 1.12).

 On the other hand, sensitivity/hurt feelings and risk-taking showed statistically negative association with self-perceived age of “older than you are.” Sensitivity/hurt feelings, suffer from “nerves,” loneliness/isolation and risk-taking showed statistically negative association with self-perceived age of “about your age.” Mood swings, miserableness, tense/highly strung’ and guilty feelings did not show statistically association with self-perceived age.

 Analysis of covariates suggested that participants who were male, younger, of low socioeconomic status (including income, education, and employment), smokers, drinkers and less active tended to be more likely to have a “older” perceived age (Table S4).

###  Analysis of Excluded and Included Participants

 A total of 154 576 participants were excluded from this research duo to missing value and other answers (“prefer not to answer” and “do not know”). Compared with the included participants, participants who were excluded had a higher proportion of male and young and middle-aged people (aged below 60). More details can be found in Supplementary file 1 (Table S5).

## Discussion

 In this study, 347 892 participants were included to analyze the effects of negative emotions on participants’ self-perceived age, demonstrating that (1) individual experiencing negative emotions were more likely to rate them as “older than you are”; (2) the impact of different negative emotions are different, with irritability having the greatest effect on self-perceived age; (3) the results of covariates showed that gender, lifestyle factors were also the impact factors for self-perceived age.

 Self-perceived age, an indicator reflecting health status, has been used to indicate one’s health status in several studies.^27,28^ Due to its important role, scientists have been attempting to identify its influence factors, like younger age, low socioeconomic status, gender, unhealthy marriage and poor self-rated health, associating with older self-perceived age. What’s more, given that this judgement was made usually via our face, therefore, those factors accelerating the facial aging process may also matter, like sleep, smoking, drinking and so on. These factors served as covariates and were included in the analysis models. Based on previous researches,^6^ this work investigated the association between self-perceived age and negative emotions.

 In the present research, irritability was found positively correlated with older self-perceived age. The term, irritability, usually used to describe feelings of anger, annoyance and impatience, commonly experienced by individuals in daily life.^29^ It is linked to a diversity of physical and psychological conditions, such as depression and cardiovascular event.^30,31^ Other negative emotions, like worry or nervous feelings, fed-up feelings also showed statistically significant association with an older self-perceived age, are also common in people’s daily life. But they may not have the same impact as irritability. Lauri and Enrico proposed bodily maps of emotions using a unique topographical self-report method, showing that participants’ feeling for anger are more intense than sadness, anxiety or fear, which indicated that the changes occurring in the body when people feel different emotions may be different. On the contrary, a much similar term, perceived age, is also commonly used in researches, which is also an estimation for one’s actual age. Differently, it is usually rated by assessors, like nurses, even computers.^32,33^ Like self-perceived age, perceived age usually affected by objects’ appearance. But research also revealed that the assessors’ age, gender and expertise will influence their assessment.^32^

 An interesting phenomenon was observed in the study that the majority of participants reported that they perceived themselves as younger than they are (about 74%). The same phenomenon can also be observed in other studies.^4,34^ A potential explanation of this may be the increase in life expectancy and retirement age, leading to a shift in the concepts for “mid-life” and “middle-age.” For instance, people used to describing themselves are “middle-aged” in their 30s-40s, but now may be the 50s,^4^ which makes people tend to rate themselves as younger than their actual age. This phenomenon may result in relatively less data for other options, which in turn affects the overall data analysis and interpretation. For some statistical methods, such as *t* tests, having too many participants for a particular option may limit their application, as these methods usually require that the independent and normally distributed data.

 There are two strengths in this research. Firstly, based on the conclusions drawn from previous research, it is possible that individuals may experience different bodily sensations when experiencing different types of emotions. Therefore, this study incorporates various emotional types to explore whether there are differences in the effects of different types of negative emotions on self-perceived age; secondly, compared to previous researches investigating the influential factors of self-perceived age, a large number of participants were included in this research, which improved the accuracy of estimates.

 This study highlights the profound impact of self-perceived age on individual health and societal behaviors, offering important insights for public health policy. Firstly, the younger self-perception of age is directly related to better health outcomes and reduced consumption of medical resources, suggesting that policy-makers should implement proactive health promotion and disease prevention strategies for the elderly. Additionally, raising awareness about the positive aspects of aging through education and public media can help shape healthier societal perceptions and behavior patterns, thereby reducing age-related discrimination and prejudice. Policy-makers should consider these factors and design cross-sectoral interventions to ensure that older adults maintain active and dignified roles in society. Not only would this improve their self-perception of age, but it would also enhance their quality of life.

 This research has limitations that should be considered when explaining the results and conclusion. Firstly, the causality cannot be determined due to its cross-section nature; secondly, the current dataset lacks some important covariate factors, such as self-confidence and self-efficacy, which also matters in the assessment for one’s appearance^35^; thirdly, the results cannot be extended to other races due to its majority of White people. What’s more, we observed a higher proportion of younger individuals in our excluded sample, which may be attributed to their tendency to prefer not to answer or have missing data. Considering this, our study findings may be more applicable to older age groups, with limitations in generalizing to younger populations. For example, if our study finds a significant association between negative emotions and self-perceived age, this association may be more pronounced in older age groups compared to younger individuals. Therefore, when interpreting our study results, we must carefully consider the age bias that may result from the excluded sample, ensuring our conclusions are more universally applicable and accurate.

## Conclusion

 Using data from the UK Biobank, we demonstrated that negative emotions are an important contributor in self-perceived age as well as other lifestyle factors such as ultraviolet light, lack of sleep, smoking, BMI, and so on. Therefore, the role of emotional factors should not be neglected in future studies on self-perceived age.

## Ethical issues

 All participants included in the UK Biobank have provided written informed consent before invited. Researchers do not need separate ethics approval therefore.

## Competing interests

 Authors declare that they have no competing interests.

## Supplementary files

Supplementary file 1 contains Tables S1-S5.
