# Supplementary material for: Negative Emotions Are Associated With Older Self-perceived Age: A Cross-section Study From the UK Biobank
Source: Int J Health Policy Manag. 2024 Jul 9;13:8060. doi: 10.34172/ijhpm.8060 (PMC11365080; doi:10.34172/ijhpm.8060)
Supplement: Supplementary file 1 — contains Tables S1-S5. [file ijhpm-13-8060-s001.pdf]

Article title: Negative Emotions Are Associated With Older Self-perceived Age: A Cross-section Study From the UK Biobank

Journal name: International Journal of Health Policy and Management (IJHPM)

Authors' information: Tianyi Wang[1], Shixing Feng[2], Junqi Wang[3], Hangyu Li[4], Yang Song[5], Dongran Han[4]*, Yixing Liu[1]*

[1]School of Management, Beijing University of Chinese Medicine, Beijing, China.

[2]Department of Neurology, Dongfang Hospital Beijing University of Chinese Medicine, Beijing, China.

[3]Dongzhimen Hospital Beijing University of Chinese Medicine, Beijing, China.

[4]School of Life and Science, Beijing University of Chinese Medicine, Beijing, China.

[5]School of Humanities, Beijing University of Chinese Medicine, Beijing, China.

*Correspondence to: Dongran Han; Email: handongr@gmail.com & Yixing Liu; Email: yixingliu1987@gmail.com

Citation: Wang T, Feng S, Wang J, et al. Negative emotions are associated with older self-perceived age: a cross-section study from the UK Biobank. Int J Health Policy Manag. 2024;13:8060. doi:10.34172/ijhpm.8060

Supplementary file 1

Table S1. Tests of the parallel regression assumption.*

	Model 1[a]			Model 2[b]			Model 3[c]		
	Chi2	df	P	Chi2	df	P	Chi2	df	P
Wolfe Gould	7375	16	0.000	5937	23	0.000	6791	38	0.000
Brant	4670	16	0.000	3753	23	0.000	4365	38	0.000
score	5618	16	0.000	4653	23	0.000	5332	38	0.000
likelihood ratio	3873	16	0.000	3084	23	0.000	3532	38	0.000
Wald	5244	16	0.000	4517	23	0.000	5257	38	0.000

* Five parallelism tests are listed in the table. p>0.05 is considered to pass the parallel test and an ordered logistic regression model, therefore, can be used; otherwise, Multinomial Logistic Regression would be used. a. Adjusted for age and sex.

b. Adjusted for all sociodemographic variables, including Townsend Deprivation Index, income, age, gender, and ethnicity.

c. Adjusted for all variables, including sociodemographic, body mass index (BMI), smoking, alcohol, sun exposure, physical activity, and sleep duration.

Table S2. Multicollinearity test

Variable	VIF	Tolerance
Mood swings	1.77	0.5650
Miserableness	1.62	0.6189
Irritability	1.32	0.7555
Sensitivity/hurt feelings	1.41	0.7099
Fed-up feelings	1.71	0.5862
Nervous feelings	1.61	0.6215
Worrier anxious feelings	1.48	0.6761
Tense/highly strung	1.42	0.7035
Worry too long after embarrassment	1.42	0.7066
Suffer from nerves	1.45	0.6897
Loneliness/isolation	1.26	0.7957
Guilty feelings	1.34	0.7469
Risk taking	1.09	0.9198
Age	1.42	0.7024
Sex	1.17	0.8523
Ethnic background	1.04	0.9661
Qualifications	1.06	0.9448
Current employment status	1.40	0.7150
Sleep duration	1.02	0.9791
Alcohol intake frequency	1.09	0.9137
Smoking status	1.05	0.9507
Body mass indebmi	1.08	0.9267
MET	1.03	0.9745

Table S3. Adjusted OR (95% CI) of negative emotions in three groups.

Characteristics ["No" as reference]	Model 1[a]			Model 2[b]			Model 3[c]		
	Younger than you are	Older than you are	About your age	Younger than you are	Older than you are	About your age	Younger than you are	Older than you are	About your age
	Reference	OR (95%CI)	OR (95%CI)	Reference	OR (95%CI)	OR (95%CI)	Reference	OR (95%CI)	OR (95%CI)
Mood Swings	--	1.13 (1.06-1.20)**	1.03 (1.01-1.05)**	--	1.06 (0.99-1.14)	1.01 (0.99-1.03)	--	1.04 (0.97-1.12)	1.00 (0.98-1.03)
Miserableness	--	1.08 (1.02-1.15)**	1.00 (0.98-1.02)	--	1.05 (0.98-1.13)	1.00 (0.98-1.02)	--	1.03 (0.96-1.11)	0.99 (0.97-1.01)
Irritability	--	1.40 (1.33-1.48)**	1.13 (1.11-1.16)**	--	1.48 (1.39-1.58)**	1.13 (1.11-1.16)**	--	1.44 (1.35-1.54)**	1.11 (1.09-1.14)**
Sensitivity/hurt feelings	--	0.86 (0.81-0.91)**	0.87 (0.85-0.88)**	--	0.85 (0.80-0.91)**	0.86 (0.84-0.88)**	--	0.86 (0.80-0.91)**	0.86 (0.84-0.87)**
Fed-up feelings	--	1.22 (1.15-1.30)**	1.09 (1.03-1.11)**	--	1.18 (1.10-1.27)**	1.06 (1.04-1.09)**	--	1.10 (1.03-1.19)**	1.05 (1.02-1.07)**
Nervous feelings	--	1.17 (1.09-1.25)**	1.07 (1.04-1.09)**	--	1.08 (1.00-1.17)	1.07 (1.04-1.10)**	--	1.15 (1.06-1.25)**	1.09 (1.06-1.12)**
Worrier / anxious feelings	--	1.11 (1.05-1.18)**	1.08 (1.06-1.10)**	--	1.15 (1.07-1.22)**	1.07 (1.05-1.10)**	--	1.18 (1.10-1.26)**	1.09 (1.07-1.12)**
Tense / 'highly strung'	--	1.15 (1.08-1.23)**	0.95 (0.93-0.98)**	--	1.08 (1.00-1.17)*	0.97 (0.94-1.00)*	--	1.06 (0.98-1.15)	0.98 (0.95-1.01)
Worry too long after embarrassment	--	1.06 (1.00-1.12)*	1.05 (1.03-1.07)**	--	1.14 (1.07-1.22)**	1.05 (1.03-1.08)**	--	1.19 (0.92-1.08)**	1.06 (1.04-1.08)**
Suffer from 'nerves'	--	1.05 (0.99-1.12)	0.98 (0.96-1.00)	--	0.99 (0.92-1.07)	0.95 (0.93-0.98)**	--	1.00 (0.92-1.08)	0.95 (0.92-0.97)**
Loneliness/ isolation	--	1.30 (1.22-1.38)**	0.87 (0.85-0.89)**	--	1.16 (1.08-1.25)**	0.88 (0.85-0.90)**	--	1.08 (1.00-1.17)*	0.86 (0.84-0.89)**
Guilty feelings	--	1.05 (0.99-1.11)	0.99 (0.97-1.01)	--	1.07 (1.00-1.14)	1.00 (0.98-1.03)	--	1.04 (0.97-1.12)	1.00 (0.98-1.02)
Risk-taking	--	0.92 (0.88-0.97)**	0.75 (0.74-0.76)**	--	0.93 (0.88-0.99)*	0.77 (0.75-0.78)**	--	0.89 (0.84-0.95)**	0.76 (0.74-0.78)**

a. Adjusted for age and sex.

b. Adjusted for all sociodemographic covariates, including Townsend Deprivation Index, income, age, gender, and ethnicity.

c. Adjusted for all covariates, including sociodemographic, body mass index (BMI), smoking, alcohol, sun exposure, physical activity, and sleep duration.

* The results were significant at the 0.05 level

** The results were significant at the 0.01 level

Table S4. Adjusted OR (95% CI) of covariates in three groups.

Characteristics	Model 1[a]			Model 2[b]			Model 3[c]		
	Younger than you are<br>Reference	Older than you are<br>OR (95%CI)	About your age<br>OR (95%CI)	Younger than you are<br>Reference	Older than you are<br>OR (95%CI)	About your age<br>OR (95%CI)	Younger than you are<br>Reference	Older than you are<br>OR (95%CI)	About your age<br>OR (95%CI)
Sex, female as reference male	--	5.36<br>(5.06-5.67)**	1.59<br>(1.56-1.62)**	--	6.26<br>(5.83-6.71)**	1.68<br>(1.65-1.71)**	--	6.22<br>(5.78-6.71)**	1.63<br>(1.60-1.66)**
Age at recruitment, 39-49 as reference									
50-59<br>(0.63-0.72)**	--	0.73<br>(0.69-0.77)**	0.90<br>(0.88-0.92)**	--	0.67	0.83<br>(0.81-0.85)**	--	0.65<br>(0.61-0.69)**	0.82<br>(0.80-0.83)**
		0.34	0.84	--	0.26<br>(0.24-0.28)**	0.64<br>(0.62-0.66)**	--	0.26<br>(0.24-0.29)**	0.63<br>(0.61-0.64)**
60-73	--	(0.32-0.37)**	(0.82-0.85)**						
Townsend deprivation index at recruitment, the first 25th percentile as reference									
2	--	--	--	--	1.00<br>(0.92-1.08)	0.97<br>(0.95-1.00)*	--	0.97<br>(0.89-1.05)	0.97<br>(0.95-1.00)*
3	--	--	--	--	0.94<br>(0.87-1.02)				
4(most deprived)	--	--	--	--	0.96<br>(0.88-1.04)				

						0.93 (0.90-0.95)**		0.89 (0.82-0.97)**	0.91 (0.89-0.94)**
						0.82 (0.80-0.84)**		0.82 (0.76-0.90)**	0.79 (0.77-0.81)**
Qualifications, College or University degree as reference									
Other academic qualifications	--	--	--	--	1.04 ()0.98-0.11)	1.02 (1.00-1.04)*	--	0.96 (0.90-1.02)	1.00 (0.98-1.02)
Current employment status, in employment as reference									
Unemployment	--	--	--	--	1.40 (1.30-1.51)**	1.37 (1.34-1.41)**	--	1.37 (1.26-1.48)**	1.38 (1.35-1.42)**
Ethnic background, White as reference									
Mixed	--	--	--	--	1.26 (1.11-1.43)**	0.97 (0.92-1.02)	--	1.16 (1.02-1.33)*	0.96 (0.91-1.01)
Asian	--	--	--	--	0.81 (0.69-0.94)**	0.76 (0.72-0.80)**	--	0.78 (0.67-0.92)**	0.75 (0.71-0.79)**
Black African	--	--	--	--	0.80 (0.55-1.19)	0.54 (0.46-0.64)**	--	0.77 (0.52-1.14)	0.54 (0.46-0.64)**
Chinese	--	--	--	--	0.59 (0.31-1.11)	0.42 (0.46-0.64)**	--	0.62 (0.33-1.17)	0.44 (0.34-0.55)**
Other	--	--	--	--	1.06 (0.80-1.40)	0.53 (0.46-0.61)**	--	0.90 (0.67-1.21)	0.51 (0.44-0.59)**
Body mass index (BMI), ≤ 24.9 as reference (kg/m^2)									
≤ 24.9	--	--	--	--	--	--	--	0.31 (0.23-0.40)**	0.82 (0.75-0.91)**
24.9 – 29.9	--	--	--	--	--	--	--	0.37 (0.27-0.50)**	0.89 (0.78-1.02)

--

--

≥ 29.9	--	--	--	--	--	--	--	0.71 (0.52-0.97)*	1.17 (1.02-1.34)*
Sleep duration, < 7h as reference									
7h – 8h	--	--	--	--	--	--	--	0.86 (0.81-0.92)**	1.07 (1.04-1.09)**
> 8h	--	--	--	--	--	--	--	1.06 (0.94-1.19)	1.06 (1.02-1.10)**
Smoking status, Never as reference									
Previous	--	--	--	--	--	--	--	1.06 (1.00-1.13)	1.03 (1.01-1.05)**
Current	--	--	--	--	--	--	--	1.60 (1.47-1.73)**	1.20 (1.16-1.24)**
Alcohol intake frequency, Daily or almost daily as reference									
Three or four times a week	--	--	--	--	--	--	--	0.94 (0.86-1.02)	0.98 (0.96-1.01)
Once or twice a week	--	--	--	--	--	--	--	0.98 (0.90-1.06)	0.93 (0.91-0.96)**
One to three times a month	--	--	--	--	--	--	--	1.05 (0.95-1.17)	0.93 (0.90-0.97)**
Special occasions only	--	--	--	--	--	--	--	1.30 (1.17-1.45)**	0.97 (0.94-1.01)
Never	--	--	--	--	--	--	--	1.55 (1.38-1.74)**	1.07 (1.02-1.12)**
Total MET minutes, <396 minutes as reference (minutes)									
396-1398	--	--	--	--	--	--	--	0.92 (0.85-0.99)*	1.04 (1.01-1.07)**
1398-3132	--	--	--	--	--	--	--	0.80 (0.74-0.87)**	0.97 (0.94-0.99)**
>3132	--	--	--	--	--	--	--	0.75 (0.69-0.81)**	0.89 (0.86-0.91)**
Time spent outdoors in summer	--	--	--	--	--	--	--	0.97 (0.96-0.99)**	0.98 (0.98-0.99)**
Time spent outdoors in winter	--	--	--	--	--	--	--	1.03 (1.01-1.05)**	1.02 (1.01-1.02)**

a. Adjusted for age and sex.

b. Adjusted for all sociodemographic covariates, including Townsend Deprivation Index, income, age, gender, and ethnicity.

c. Adjusted for all covariates, including sociodemographic, body mass index (BMI), smoking, alcohol, sun exposure, physical activity, and sleep duration. *

The results were significant at the 0.05 level

** The results were significant at the 0.01 level

Table S5. Comparison between including and excluding participants.

	Overall	Excluded	Included	P value
n	502,468	154,576	347,892	
**Sex, n (%)**				**<0.001**
Female	273,360 (54.4)	88,577 (57.3)	184,783 (53.1)	
Male	229,108 (45.6)	65,999 (42.7)	163,109 (46.9)	
Missing value	--	--	--	
**Age at recruitment, n (%)**				**<0.001**
39-49	117,853 (23.5)	33,857 (21.9)	83,996 (24.1)	
50-59	167,138 (33.3)	49,675 (32.1)	117,463 (33.8)	
60-73	217,477 (43.3)	71,044 (46.0)	146,433 (42.1)	
Missing value	--	--	--	
**Townsend deprivation index, n (%)**				**<0.001**
Q1	125,489 (25.0)	34,760 (22.6)	90,729 (26.1)	
Q2	125,455 (25.0)	36,130 (23.5)	89,325 (25.7)	
Q3	125,444 (25.0)	38,073 (24.7)	87,371 (25.1)	
Q4	125,456 (25.0)	44,989 (29.2)	80,467 (23.1)	
Missing value	624	624	--	
**Qualifications, n (%)**				**<0.001**
College or University degree	161,148 (32.7)	41,934 (29.0)	119,214 (34.3)	
Other academic qualifications	341320 (67.2)	139,715 (67.2)	228,678 (65.7)	
Missing value	10,133	10,133	--	
**Current employment status, n (%)**				**<0.001**
In employment	287,123 (57.5)	78,548 (51.8)	208,575 (60.0)	
Unemployment	212,394 (42.5)	72,337 (48.2)	139,317 (40.0)	
Missing value	2,951	2,951	--	
**Race, n (%)**				**<0.001**
White/British	454,212 (90.9)	134,781 (88.8)	319,431 (91.8)	
Mixed	18,910 (3.8)	6,912 (4.6)	11,998 (3.4)	
Asian	17,562 (3.5)	5,945 (3.9)	11,617 (3.3)	
Black African	2,875 (0.6)	1,293 (0.9)	1,582 (0.5)	
Chinese	1,574 (0.3)	692 (0.5)	882 (0.3)	
Other	4,558 (0.9)	2,176 (1.4)	2,382 (0.7)	
Missing value	2,777 (0.0)	2,777 (0.0)	--	
**BMI, n (%)**				**<0.001**
< 24.9	160,037 (31.9)	47,830 (30.9)	112,207 (32.3)	
25 – 29.9	214,201 (42.6)	63,103 (40.8)	151,098 (43.4)	
≥ 30	128,230 (25.5)	43,643 (28.2)	84,587 (24.3)	
Missing value	--	--	--	
**Sleep duration, n (%)**				**<0.001**

< 7h	123,241 (24.5)	40,842 (26.4)	82,399 (23.7)	
7h–8h	336,667 (67.0)	97,611 (63.1)	239,056 (68.7)	
> 8h	42,560 (8.5)	16,123 (10.4)	26,437 (7.6)	
Missing value	--	--	--	
**Smoking status, n (%)**				**<0.001**
Never	273,501 (54.8)	82,612 (54.5)	190,889 (54.9)	
Previous	173,045 (34.6)	51,317 (33.8)	121,728 (35.0)	
Current	52,973 (10.6)	17,698 (11.7)	35,275 (10.1)	
Missing value	2,949 (0.0)	2,949 (0.0)	--	
**Alcohol intake frequency, n (%)**				**<0.001**
Daily or almost daily	101,766 (20.3)	27,425 (17.9)	74,341 (21.4)	
Three or four times a week	115,432 (23.0)	32,272 (21.1)	83,160 (23.9)	
Once or twice a week	129,284 (25.8)	38,774 (25.3)	90,510 (26.0)	
One to three times a month	55,848 (11.1)	17,645 (11.5)	38,203 (11.0)	
Special occasions only	58,002 (11.6)	21,212 (13.9)	36,790 (10.6)	
Never	40,635 (8.1)	15,747 (10.3)	24,888 (7.2)	
Missing value	1,501 (0.0)	1,501 (0.0)	--	
**MET score, n (%)**				**<0.001**
Q1	147,318 (29.3)	68,520 (44.3)	78,798 (22.7)	
Q2	120,230 (23.9)	30,807 (19.9)	89,423 (25.7)	
Q3	117,873 (23.5)	28,061 (18.2)	89,812 (25.8)	
Q4	117,047 (23.3)	27,188 (17.6)	89,859 (25.8)	
Missing value	--	--	--	
**Summer time, median [25th, 75th]**	3.00 [2.00, 5.00]	3.00 [2.00, 5.00]	3.00 [2.00, 5.00]	<0.001
**Winter time, median [25th, 75th]**	1.00 [1.00, 2.00]	1.00 [1.00, 2.00]	1.00 [1.00, 2.00]	0.479
**Facial aging, n (%)**				**<0.001**
Younger than you are	338,652 (68.0)	80,929 (54.0)	257,723 (74.1)	
Older than you are	9,609 (1.9)	2,472 (1.6)	7,137 (2.1)	
About your age	108,923 (21.9)	25,891 (17.3)	83,032 (23.9)	
Prefer not to answer	525 (0.1)	525 (0.4)	0 (0.0)	
Do not know	40,088 (8.1)	40,088 (26.7)	0 (0.0)	
Missing value	4,671	4,671	--	
**Mood swings, n (%)**				**<0.001**
No	265,652 (53.0)	67,358 (43.8)	198,294 (57.0)	
Yes	222,508 (44.4)	72,910 (47.4)	149,598 (43.0)	
Prefer not to answer	964 (0.2)	964 (0.6)	0 (0.0)	
Do not know	12,439 (2.5)	12,439 (8.1)	0 (0.0)	
Missing value	905	905	--	
**Miserableness, n (%)**				**<0.001**
No	281,484 (56.1)	76,790 (50.0)	204,694 (58.8)	
Yes	210,590 (42.0)	67,392 (43.9)	143,198 (41.2)	
Prefer not to answer	1,032 (0.2)	1,032 (0.7)	0 (0.0)	
Do not know	8,457 (1.7)	8,457 (5.5)	0 (0.0)	
Missing value	905	905	--	
**Irritability, n (%)**				**<0.001**
No	344,053 (68.6)	91,254 (59.4)	252,799 (72.7)	

Yes	133,892 (26.7)	38,799 (25.2)	95,093 (27.3)	
Prefer not to answer	1,266 (0.3)	1,266 (0.8)	0 (0.0)	
Do not know	22,352 (4.5)	22,352 (14.5)	0 (0.0)	
Missing value	905	905	--	
**Sensitivity hurt feelings, n (%)**				**<0.001**
No	216,475 (43.2)	55,601 (36.2)	160,874 (46.2)	
Yes	269,666 (53.8)	82,648 (53.8)	187,018 (53.8)	
Prefer not to answer	1,062 (0.2)	1,062 (0.7)	0 (0.0)	
Do not know	14,359 (2.9)	14,359 (9.3)	0 (0.0)	
Missing value	906	906	--	
**Fed up feelings, n (%)**				**<0.001**
No	290,777 (58.0)	76,484 (49.8)	214,293 (61.6)	
Yes	199,127 (39.7)	65,528 (42.6)	133,599 (38.4)	
Prefer not to answer	1,376 (0.3)	13,76 (0.9)	0 (0.0)	
Do not know	10,282 (2.0)	10,282 (6.7)	0 (0.0)	
Missing value	906	906	--	
**Nervous feelings, n (%)**				**<0.001**
No	372,355 (74.2)	100,835 (65.6)	271,520 (78.0)	
Yes	115,184 (23.0)	38,812 (25.3)	76,372 (22.0)	
Prefer not to answer	793 (0.2)	793 (0.5)	0 (0.0)	
Do not know	13,230 (2.6)	13230 (8.6)	0 (0.0)	
Missing value	906	906	--	
**Worrier anxious feelings, n (%)**				**<0.001**
No	212,072 (42.3)	51,344 (33.4)	160,728 (46.2)	
Yes	275,514 (54.9)	88,350 (57.5)	187,164 (53.8)	
Prefer not to answer	1,026 (0.2)	1,026 (0.7)	0 (0.0)	
Do not know	12,949 (2.6)	12,949 (8.4)	0 (0.0)	
Missing value	906	906	--	
**Tense highly strung, n (%)**				**<0.001**
No	396,917 (79.1)	108,065 (70.3)	288,852 (83.0)	
Yes	85,721 (17.1)	26,681 (17.4)	59,040 (17.0)	
Prefer not to answer	979 (0.2)	979 (0.6)	0 (0.0)	
Do not know	17,944 (3.6)	17,944 (11.7)	0 (0.0)	
Missing value	907	907	--	
**Worry too long, n (%)**				**<0.001**
No	251,061 (50.1)	67,410 (43.9)	183,651 (52.8)	
Yes	229,071 (45.7)	70,500 (45.9)	158,571 (45.6)	
Prefer not to answer	992 (0.2)	963 (0.6)	29 (0.0)	
Do not know	20,437 (4.1)	14,796 (9.6)	5,641 (1.6)	
Missing value	907	907	--	
**Suffer from nerves, n (%)**				**<0.001**
No	379,925 (75.7)	103,112 (67.1)	276,813 (79.6)	

Yes	101,917 (20.3)	30,838 (20.1)	71,079 (20.4)	
Prefer not to answer	1,041 (0.2)	1,041 (0.7)	0 (0.0)	
Do not know	18,678 (3.7)	18,678 (12.2)	0 (0.0)	
Missing value	907	907	--	
**Loneliness/isolation, n (%)**				**<0.001**
No	401,205 (80.0)	114,193 (74.3)	287,012 (82.5)	
Yes	91,394 (18.2)	30,514 (19.9)	60,880 (17.5)	
Prefer not to answer	1,430 (0.3)	1,430 (0.9)	0 (0.0)	
Do not know	7,531 (1.5)	7,531 (4.9)	0 (0.0)	
Missing value	908	908	--	
**Guilty feelings, n (%)**				**<0.001**
No	346,524 (69.1)	96,167 (62.6)	250,357 (72.0)	
Yes	140,548 (28.0)	43,013 (28.0)	97,535 (28.0)	
Prefer not to answer	1,633 (0.3)	1,633 (1.1)	0 (0.0)	
Do not know	12,855 (2.6)	12,855 (8.4)	0 (0.0)	
Missing value	908	908	--	
**Risk taking, n (%)**				**<0.001**
No	352,197 (70.2)	102,867 (66.9)	249,330 (71.7)	
Yes	129,834 (25.9)	31,272 (20.4)	98,562 (28.3)	
Prefer not to answer	1,025 (0.2)	1,025 (0.7)	0 (0.0)	
Do not know	18,504 (3.7)	18,504 (12.0)	0 (0.0)	
Missing value	908	908	--	

**Data were expressed by number and percentage or median and 25th and 75th quantiles .
